# A Different Pattern of Arrangement of the Risorius Muscle Fibers: A Case Report

**DOI:** 10.7759/cureus.22922

**Published:** 2022-03-07

**Authors:** Beatriz C Ferreira-Pileggi, Alexandre R Freire, Paulo R Botacin, Felippe B Prado, Ana Cláudia Rossi

**Affiliations:** 1 Biosciences, Piracicaba Dental School, University of Campinas (UNICAMP), Piracicaba, BRA; 2 Biosciences/Anatomy, Piracicaba Dental School, University of Campinas (UNICAMP), Piracicaba, BRA; 3 Basic Sciences, Araçatuba Dental School, Paulista State University (UNESP), Araçatuba, BRA

**Keywords:** face, morphology, macroscopic human anatomy, risorius muscle, facial expression muscles

## Abstract

The risorius is a very thin muscle and is one of the superficial muscles of facial expression, which is reported as being inconstant, as it is absent in most people, and is a unique muscle; once it has no bony origin. The study aimed to report a different pattern of arrangement of the risorius muscle fibers. During a routine Anatomy class, a different pattern of arrangement of the risorius muscles fibers was found from the observation of an adult (around 40 years old) male cadaver hemiface (right side). After dissections, it was possible to note that the arrangement of the risorius muscle fibers on the surface of the masseteric fascia was formed in different arrangements formed in two bands. In conclusion, the risorius muscle fibers are not always easily distinguishable, and detailed knowledge of their different morphological arrangement is essential when planning and performing facial procedures.

## Introduction

The facial expression muscles are significantly important to human behavior due to their contribution to a wide range of functions, for example, feeding, speech, and communication of affective states [1]. They are known for presenting important anatomical variability, including its shape, size, origin, and insertion pattern and its presence or absence [2-5]. 

According to Waller et al. [2], the muscles that are essential for universal facial expression present little variation among individuals, while the muscles that are nonessential for the occurrence of universal facial expression exhibit great asymmetry and inconsistency in presence. The knowledge of the anatomy of the risorius muscle is important as a reference for injections of botulinum neurotoxin Type A. Bae et al. [6] dissected 48 hemifaces. The locations of origin and insertion points of the risorius muscle were measured, and the masseter muscle was divided into six equally sized rectangular areas. The authors suggested that the medial part of the masseter muscle represents a hazard zone into which the injection of botulinum neurotoxin Type A may affect the risorius muscle, potentially resulting in iatrogenic unnatural facial expressions.

The risorius is a very thin muscle and is one of the superficial muscles of facial expression, which is reported as being inconstant, as it is absent in most people and is a unique muscle; it has no bony origin [4,7-8]. It becomes narrower from its origin, in the fascia of the lateral cheek over the parotid gland, the superficial masseter muscle, and the platysma muscle, to its insertion, at the skin of the mouth angle [4,9]. The risorius muscle is responsible for retracting the mouth angle, enabling the facial expression of smiling and laughing [4,9]. 

The present study aims to report a different pattern of arrangement of the risorius muscle fibers to contribute with surgical planning and performing of facial procedures like the reconstructive ones, the facial reanimation operation, and the cosmetic surgeries as well, like those aiming to correct developmental defects, facial traumas, facial muscle paralysis and restoring the natural personal appearances [4-5,9-10]. 

## Case presentation

During a routine Anatomy class, a different pattern of arrangement of the risorius muscles fibers was found from the observation of an adult (around 40 years old) male cadaver hemiface (right side), fixed in 10% formalin, in the Anatomy Laboratory of the Araçatuba Dental School, Paulista State University (UNESP), São Paulo, Brazil. 

**Figure 1:**
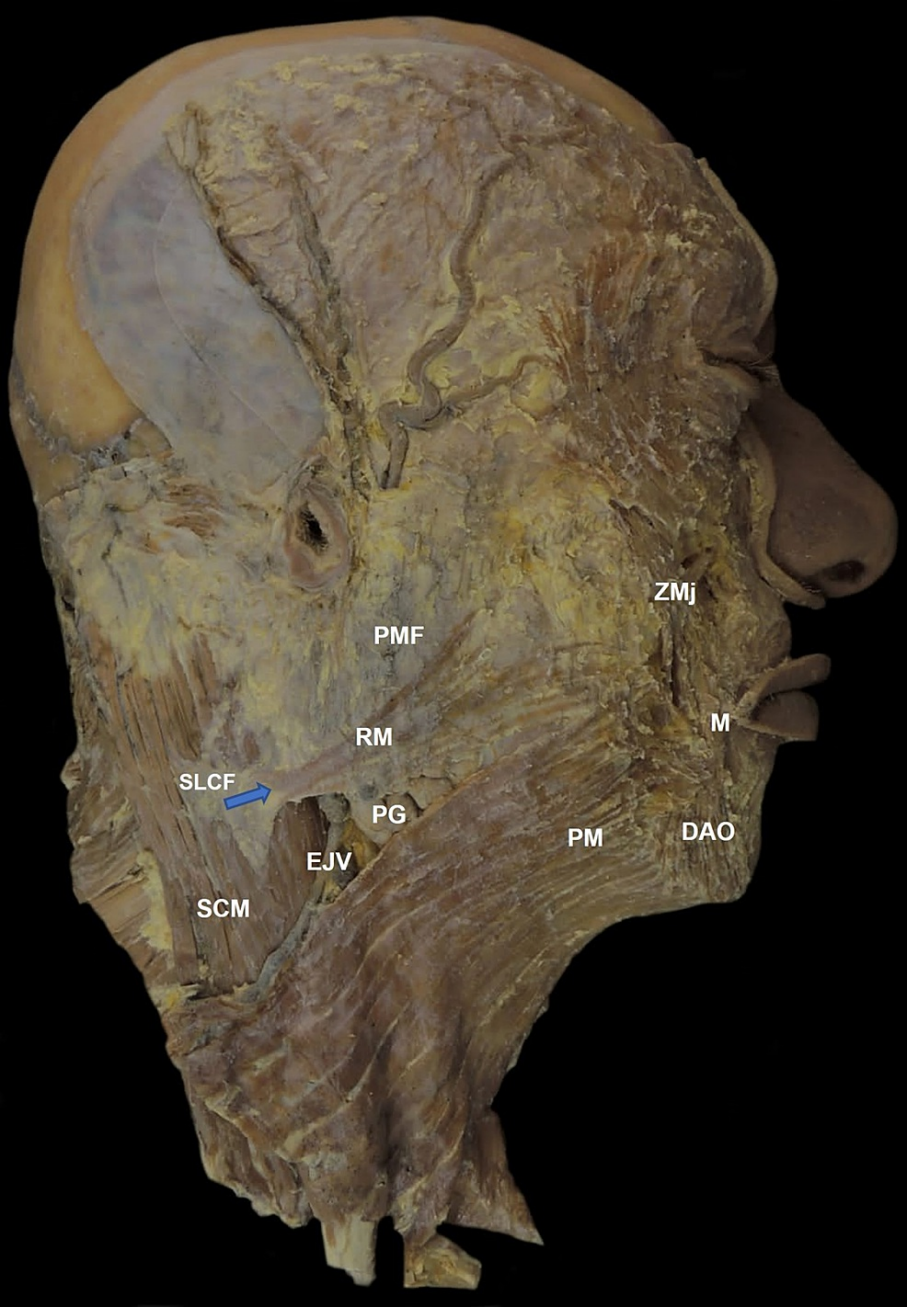
Lateral (right side) view of the face The blue arrow shows the risorius muscle origin. SLCF: superficial layer of cervical fascia. SCM: sternocleidomastoid muscle. EJV: external jugular vein. PG: parotid gland. RM: risorius muscle. PMF: parotid-masseteric fascia. PM: platysma muscle. DAO: depressor anguli oris muscle. M: modiolus. ZMj: zygomatic major muscle.

A layered dissection using the outside-in technique was performed: after removal of the skin and the subcutaneous fat, it was curious to note that the arrangement of the risorius muscle fibers on the surface of the masseteric fascia was formed in two bands. In the first band, the origin of the fiber started at the superficial layer of the cervical fascia of the sternocleidomastoid muscle, at the level of the mandible angle, inserting into the surface of the parotid-masseteric fascia in a fan-shaped distribution, noting also that some fibers meet with the fibers of the platysma muscle (Figure 1).

**Figure 2:**
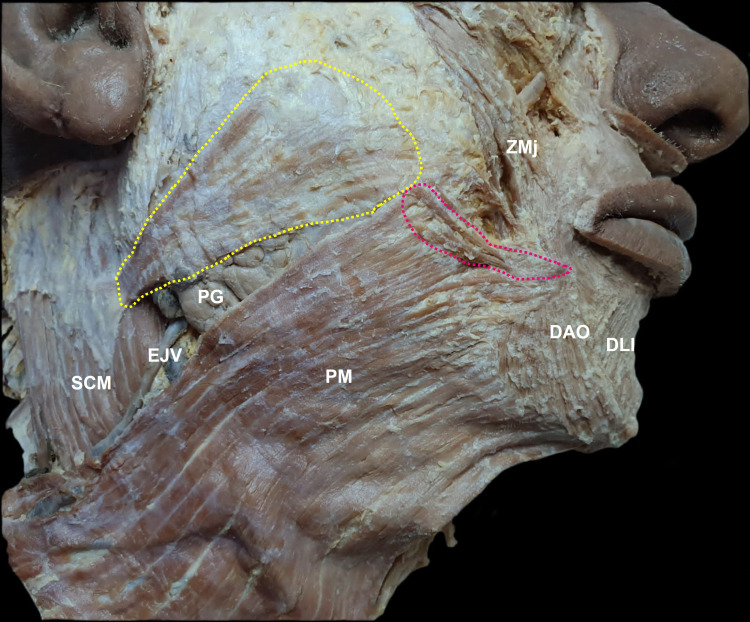
Close-up right-side view of the face. The dashed lines show the two bands of fibers of the risorius muscle. The yellow dashed lines show the first band of fibers. The pink dashed lines show the second band of fibers. SCM: sternocleidomastoid muscle. EJV: external jugular vein. PG: parotid gland. PM: platysma muscle. DAO: depressor anguli oris muscle. DLI: depressor labii inferioris muscle. ZMj: zygomatic major muscle.

At the level of the anterior border of the masseter muscle (at the height of the angle of the mouth), was verified that the second band fibers emerge, which seems to be continuous fibers with the anterior ones (that are distributed on the surface of the parotid-masseteric fascia) and that are directed to insertion in the modiolus (Figure 2). At this point, the risorius fibers mixed with the fibers from the depressor anguli oris and the zygomatic major muscles. This second band also has fibers that meet with the platysma muscle. The pattern of the risorius muscle on the left side was a single band that the fibers are distributed on the surface of the parotid-masseteric fascia until the insertion in the modiolus.

## Discussion

During aesthetics procedures, i.e., facelift, the superficial musculoaponeurotic system (SMAS) layer is surgically dissected, and the facial muscles are manipulated through various flap dissections and surgical approaches. Special attention is required when performing dissections at the level of the parotid-masseteric region to avoid injuring the branches of the facial nerve, masseteric ligament, and muscle fibers of the risorius [10-11]. 

The anatomy of the risorius muscle presents a high variability, as it can range from one or more thin fascicles to a single, wide band of muscle [12]. Bae et al. [12] described different origins of this muscle from the superficial muscular aponeurotic system (SMAS) layer, the parotid-masseteric fascia, or the masseter tendon. Moreover, the authors found that some risorius fibers can be located with the facial nerve and the parotid duct within the deep fascial plane of the face, and other fibers may lie along with the platysma muscle within the SMAS plane [12]. 

Waller et al. [2] analyzed 18 adult human cadavers and reported that the risorius muscle because it is not an essential muscle, showed significant variation among individuals regarding bilateral symmetry, asymmetry, and its presence or absence [2]. Despite that, Germann and Al Khalili [8] reported that in most individuals in which the risorius muscle is present (incidence of risorius is 95.8%), it is located on either side of the cheeks. 

In the present study, the arrangement of the risorius muscle fibers on the surface of the masseteric fascia was formed in two bands. In the literature, some authors reported different arrangements of the risorius muscles, such as Kim et al. [9]. The authors made a study of the arrangements of the risorius muscle, in which they dissected 80 hemifaces of Asian cadavers. They reported that, in this population, the risorius muscle is found in three different types, depending on the insertion patterns, such as the zygomaticus risorius, the platysma risorius, and the triangularis risorius [9]. They also exposed that those different types of risorius may permit different functions of the muscle. It may act retracting the labial commissures, or be part of the lip elevators, or may not have any elevating effects on the lips, or it may even help depress the mouth angle downwards, forming an expression of sorrow [13]. 

It is possible to note in the literature the topographic anatomy of the risorius muscle and the anatomical relationships with other muscles present in the face. The risorius muscle is described as superficial to the orbicularis oris and the depressor anguli oris muscles at its most anterior portion, and superficial to the masseter muscle (superficial portion) at its posterior portion [8]. In the present report, the risorius muscle was presented in two bands, one relating to the sternocleidomastoid and the platysma muscles, and the other band relating to the platysma, the depressor anguli oris, and the zygomatic major muscles and the modiolus. 

## Conclusions

In conclusion, in the present case report, the risorius muscle fibers on the surface of the masseteric fascia were formed in two bands. As the risorius muscle presents a high variability, its fibers are not always easily distinguishable, and detailed knowledge of their different morphological arrangement is essential when planning and performing facial procedures. 
